# Supplementary material for: A methodology for the design of experiments in computational intelligence with multiple regression models
Source: PeerJ. 2016 Dec 1;4:e2721. doi: 10.7717/peerj.2721 (PMC5136129; doi:10.7717/peerj.2721)
Supplement: Data S1 — Datailed results from UC Irvine Machine Learning Repository (Housing, Machine CPU, Wine Quality, Automobile and Parkinson) and the 3 Use Cases (Protein Corona, Gajewicz Metal Oxides and Aquatic Toxicity) [file peerj-04-2721-s001.zip › 6_UseCases/1_ProteinCorona/RFRFE.details.csv.repeatedcv.split6.pdf]

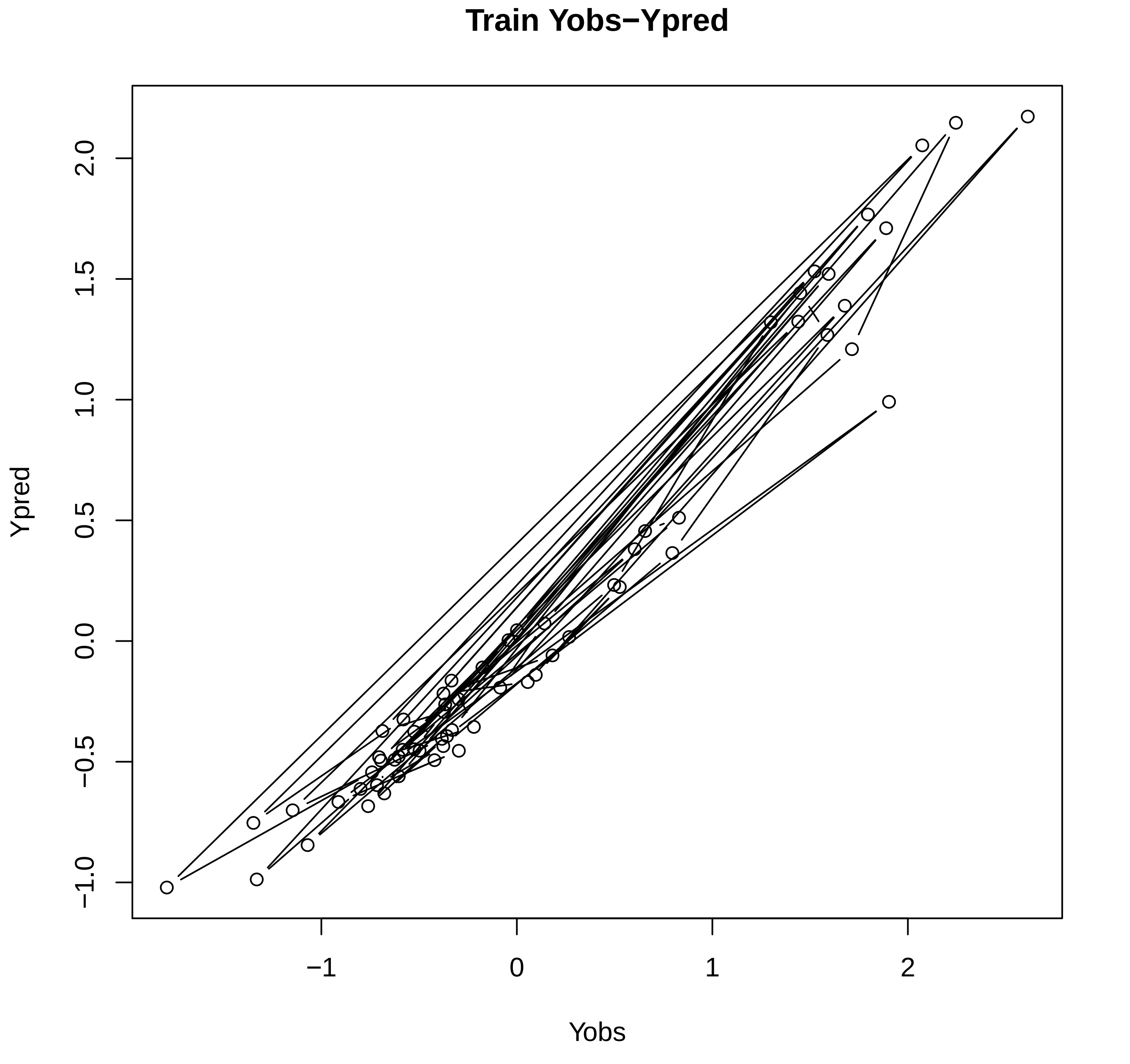

Train Yobs−Ypred
Ypred
Yobs
−1.0
−0.5
0.0
0.5
1.0
1.5
2.0
−1
0
1
2

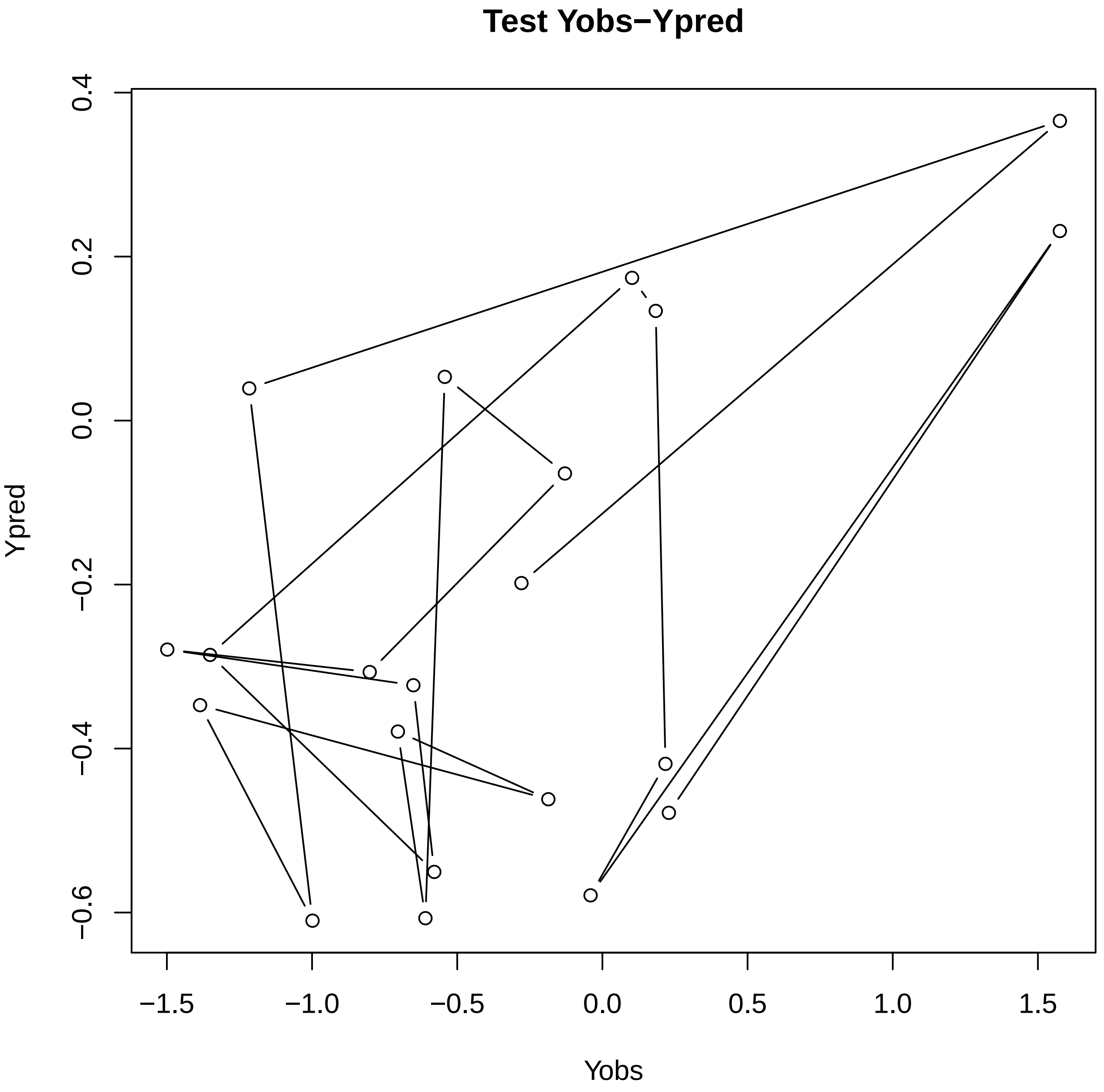

Test Yobs−Ypred
Ypred
Yobs
0.4
0.2
0.0
−0.2
−0.4
−0.6
−1.5
−1.0
−0.5
0.0
0.5
1.0
1.5

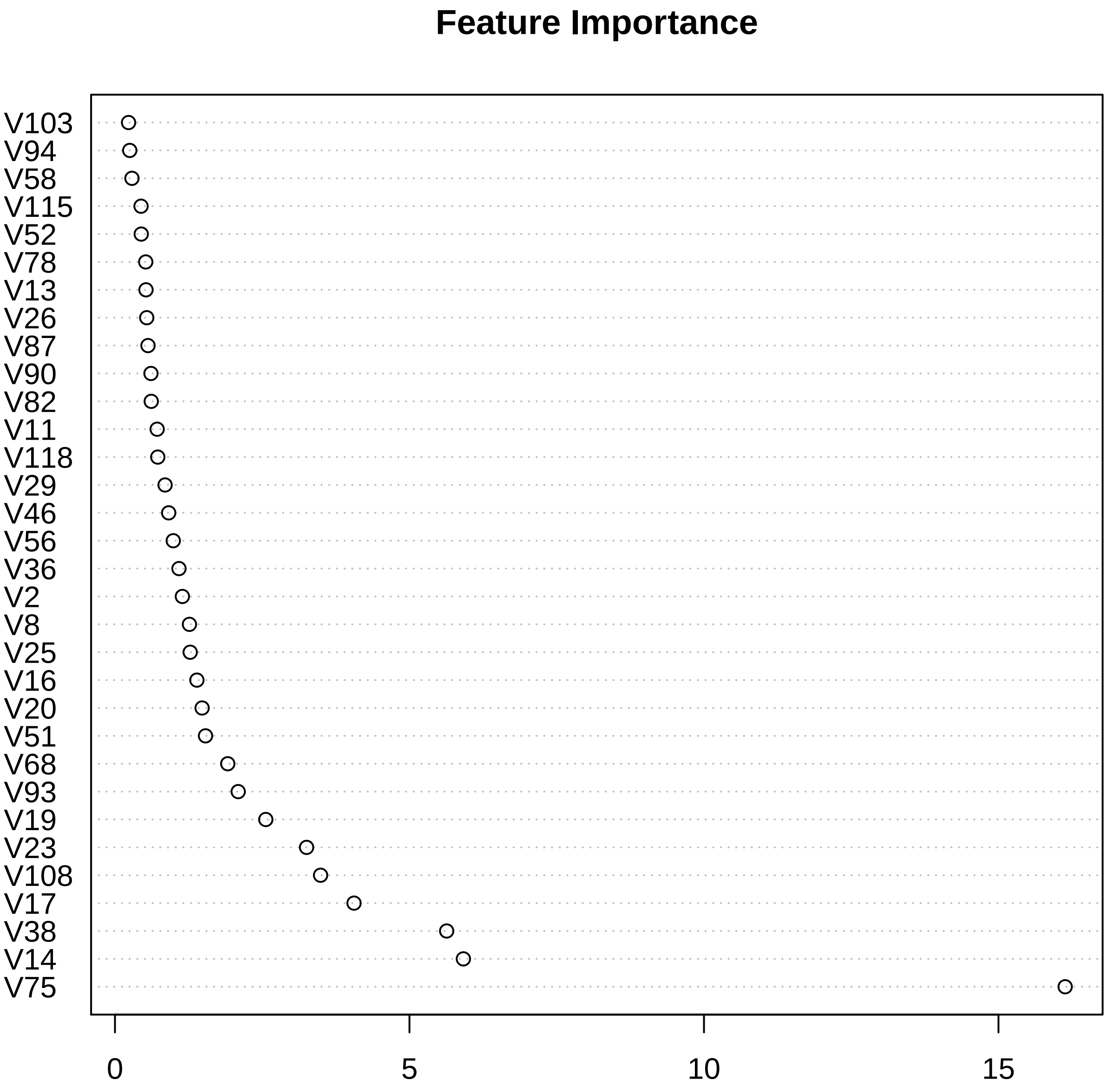

Feature Importance
V103
V94
V58
V115
V52
V78
V13
V26
V87
V90
V82
V11
V118
V29
V46
V56
V36
V2
V8
V25
V16
V20
V51
V68
V93
V19
V23
V108
V17
V38
V14
V75
0
5
10
15

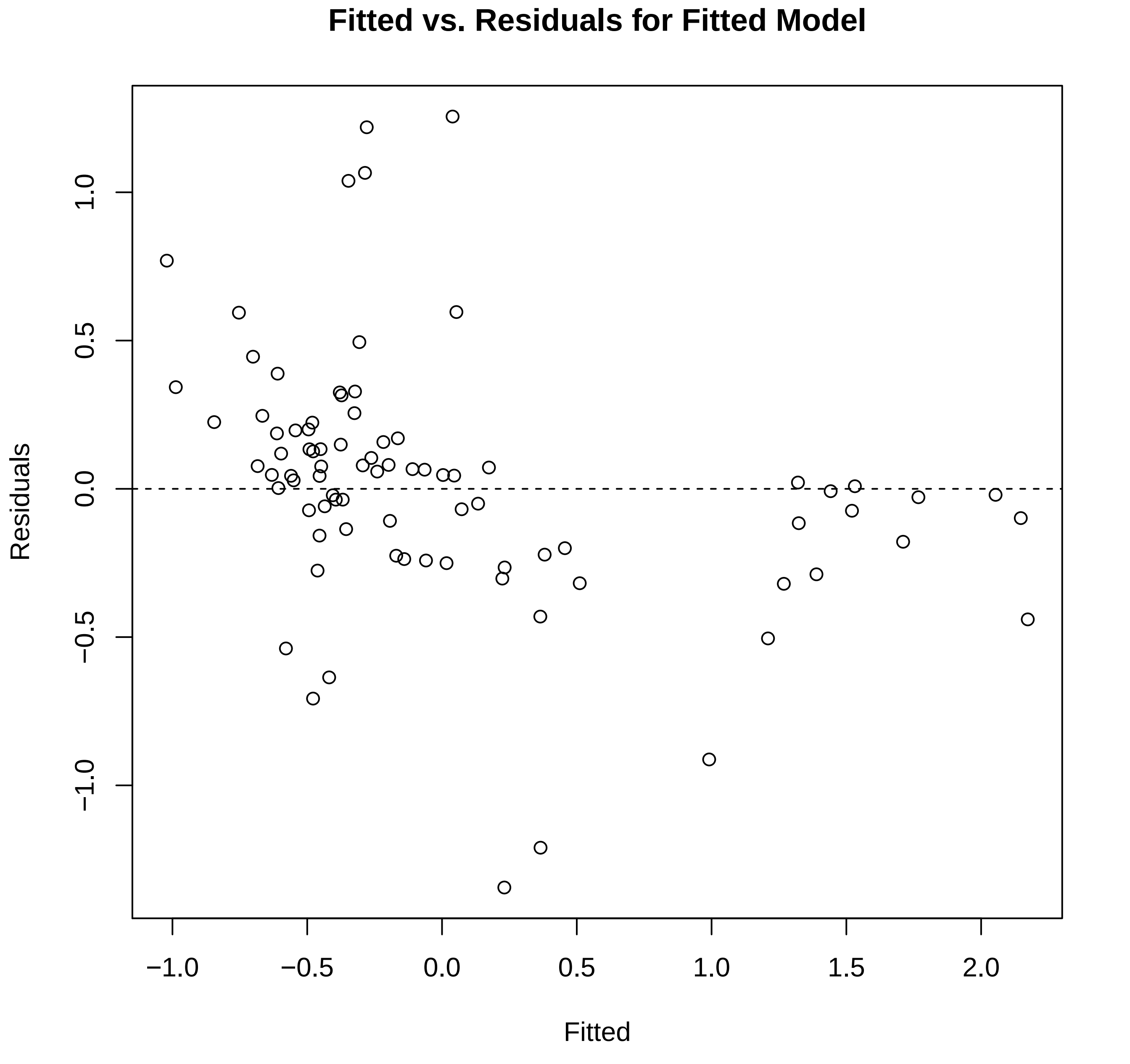
Fitted vs. Residuals for Fitted Model
Residuals
1.0
0.5
0.0
−0.5
−1.0
−1.0
−0.5
0.0
0.5
1.0
1.5
2.0
Fitted
